# Sarcomatous transformation of an IDH-mutant oligodendroglioma matching to methylation class oligosarcoma despite vorasidenib use, a case report

**DOI:** 10.1093/noajnl/vdag263

**Published:** 2026-10-23

**Authors:** Lucy P Evans, Janet La, Matija Snuderl, Vyshak A Venur, Samuel N Emerson, Kristyn Galbraith

**Affiliations:** Division of Neuropathology, Department of Laboratory Medicine and Pathology, University of Washington, Seattle, Washington; Department of Neurosurgery and Neuroscience, The Permanente Medical Group, Redwood City, California; Department of Pathology, NYU Langone Medical Center, New York, New York; Division of Medical Oncology, Department of Medicine, University of Washington, Seattle, Washington; Department of Neurological Surgery, University of Washington, Seattle, Washington; Division of Neuropathology, Department of Laboratory Medicine and Pathology, University of Washington, Seattle, Washington; Division of Molecular Pathology, Department of Laboratory Medicine and Pathology, University of Washington, Seattle, Washington

**Keywords:** DNA methylation, oligodendroglioma, oligosarcoma, targeted therapy, vorasidenib

## Abstract

Sarcomatous transformation of diffuse gliomas is rare, particularly in IDH-mutant tumors. Oligosarcoma is a recently recognized methylation class of IDH-mutant, 1p/19q-codeleted gliomas with distinct histologic, molecular, and clinical features. We report a unique case of oligosarcoma arising from an IDH-mutant, 1p/19q-codeleted oligodendroglioma following documented disease progression and subsequent treatment with the IDH-inhibitor vorasidenib in the Investigating Vorasidenib in Glioma (INDIGO) trial. The recurrent tumor demonstrated spindle cell morphology, increased reticulin deposition, retained 1p/19q codeletion, and mutations in *IDH1* p. R132H, *TERT* promoter, and *TP53*. DNA methylation profiling confirmed oligosarcoma. Following radiation and chemotherapy, a second recurrence demonstrated subclonal biallelic CDKN2A/B inactivation, consistent with continued clonal evolution. This case illustrates the molecular progression underlying sarcomatous transformation and highlights the importance of repeat tissue sampling and comprehensive molecular profiling in patients with atypical disease progression.

A previously healthy 22-year-old presented with a generalized seizure in January 2020. Brain MRI demonstrated a nonenhancing, T2/Fluid-Attenuated Inversion Recovery (FLAIR) abnormality in his left temporal lobe (Figure 1A). He started on levetiracetam and dexamethasone and underwent an awake craniotomy with language mapping and subtotal resection with a small amount of residual disease in the temporal stem and insula.

**Figure 1.**
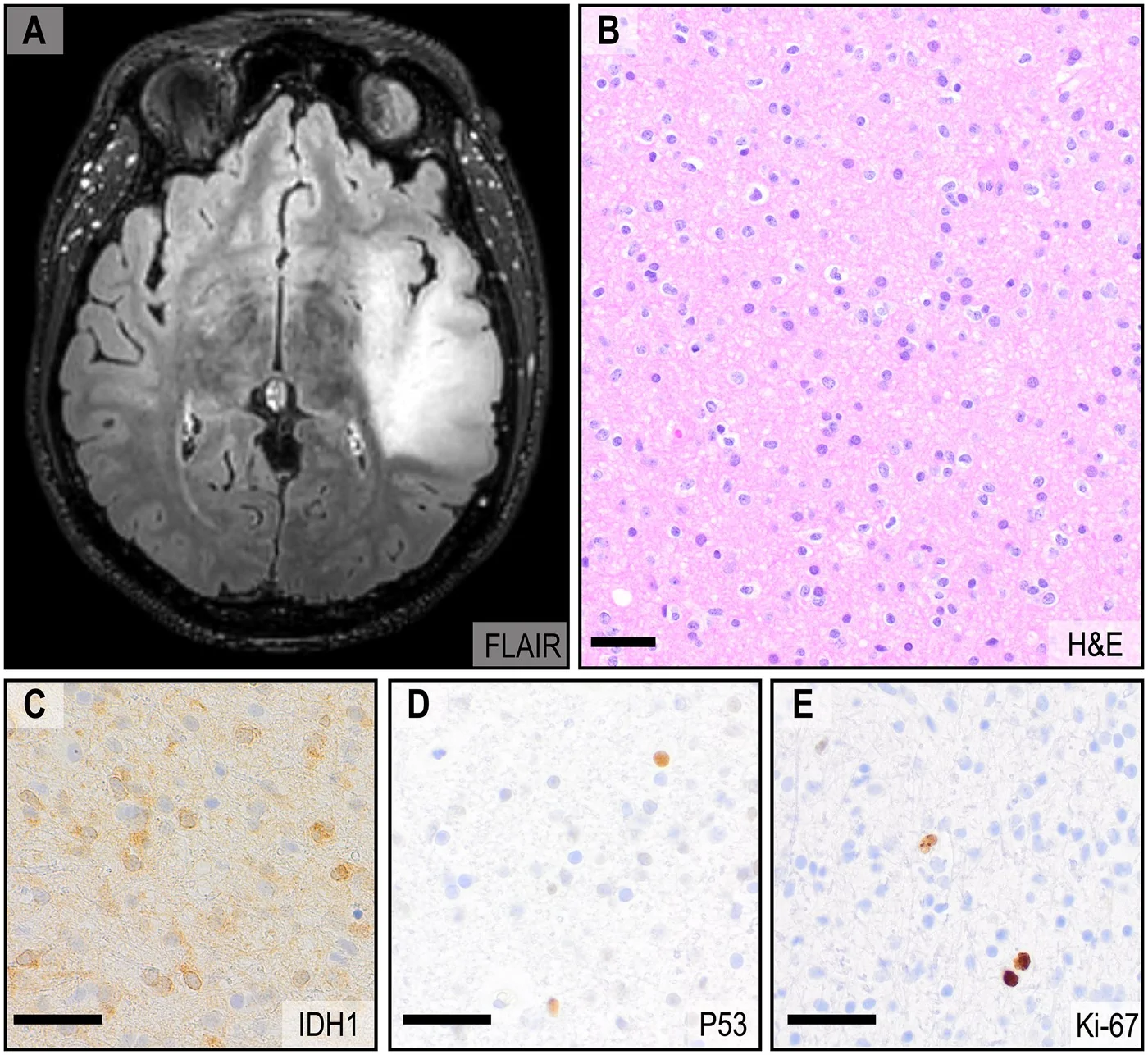
Imaging and histology of initial presentation and resection of an oligodendroglioma, IDH-mutant, CNS WHO grade 2. (A) Axial FLAIR MRI demonstrated an intrinsic, expansile, nonenhancing mass within the temporal lobe with extension into the temporal stem, insula, and subinsular white matter. (B) Hematoxylin and eosin (H&E) staining of the lesion demonstrates a low-grade infiltrating glial neoplasm with irregular nuclear contours and no necrosis or microvascular proliferation. The neoplasm demonstrates immunopositivity for (C) IDH1 R132H, (D) scattered TP53, and (E) a low proliferation index (Ki-67 of 3%). Scale bar = 20 μm.

The specimen revealed a low-grade infiltrating glioma with minimal mitotic activity and no microvascular proliferation or necrosis (Figure 1B). The neoplastic cells were immunoreactive for IDH1 R132H, a wild-type TP53 staining pattern, and a low proliferation index (Ki-67 up to 3%; Figure 1C, D, and E, respectively). Fluorescence in situ hybridization (FISH) confirmed the diagnosis of an oligodendroglioma, IDH1 (p.R132H)-mutant and 1p/19q-codeleted, CNS WHO grade 2.

Perioperatively, he had double vision and language difficulties, which resolved over several weeks with no new permanent neurologic deficits. Approximately 8 months after surgery, he developed breakthrough focal seizures consisting of olfactory changes, right-sided numbness and tingling, and impaired responsiveness without loss of consciousness, prompting an increase in levetiracetam.

He opted for observation and remained stable until enrollment 1 year later in the INDIGO clinical trial of vorasidenib, an IDH-inhibitor, versus placebo. Slow disease progression was reflected by differences between progression-free survival (PFS) and time to next treatment. The median PFS in the vorasidenib arm was 27.7 months, whereas the median time to next treatment was not reached, suggesting that providers may observe or continue vorasidenib despite slow disease progression.^1^

The patient was randomized to the placebo arm. Frequent, brief sensory seizures prompted addition of zonisamide to levetiracetam. Zonisamide was later switched to lacosamide, initially improving seizure frequency; however, seizures gradually increased.

Two years after enrollment, trial unblinding revealed radiographic disease progression, and placebo was transitioned to vorasidenib. His neurological examination remained nonfocal, but he endorsed more fatigue with initiation of vorasidenib. After 4 months on the medication, he reported a decrease in seizure frequency.

After 1.5 years on vorasidenib, imaging showed a dural-based contrast-enhancing lesion along the posterolateral resection cavity, with unchanged T2/FLAIR signal in the left temporal lobe (Figure 2A). His neurological examination remained unchanged. Short-interval MRI demonstrated definitive growth. His imaging, pathology, and clinical history were reviewed at the multidisciplinary neuro-oncology tumor board. Leading differential considerations were a meningioma versus malignant transformation. The apparent extra-axial location argued against transformation to an aggressive grade 3 (formerly known as anaplastic) oligodendroglioma or oligosarcoma, especially as his known residual in the insula had not changed. He had not had radiation, and thus a radiation-induced meningioma or cavernoma was deemed less likely.

**Figure 2.**
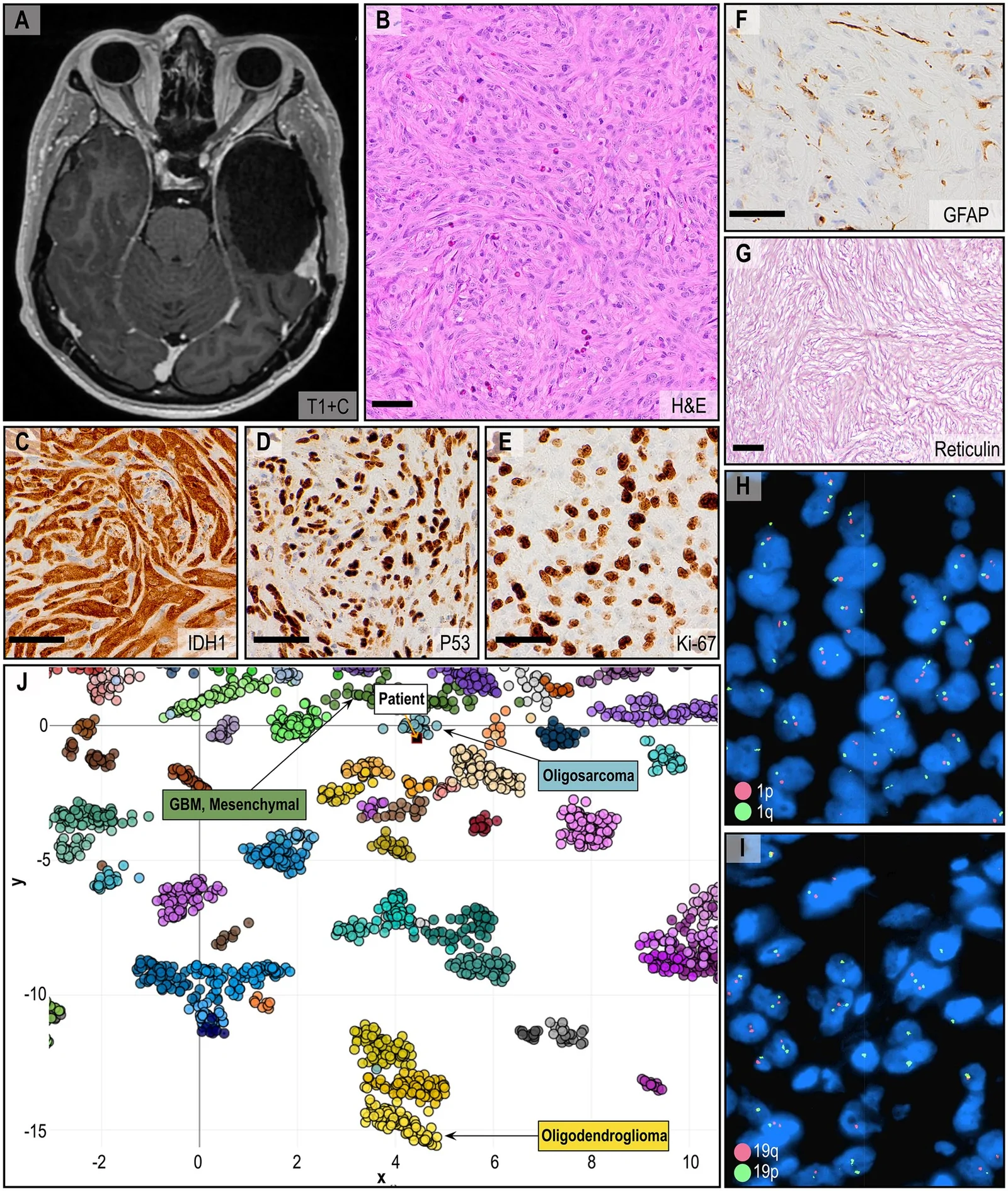
Imaging, histology, and molecular testing of recurrence of an oligodendroglioma with sarcomatous transformation. (A) Axial T2 post-contrast magnetic resonance imaging of an enhancing extra-axial mass at the posterior margin of the resection cavity with interval growth on subsequent imaging. (B) Hematoxylin and eosin (H&E) staining of the recurrence demonstrates a highly cellular, spindle cell neoplasm with marked nuclear pleomorphism and focal necrosis. The neoplastic cells demonstrate immunopositivity for (C) IDH1 R132H, (D) increased TP53, and (E) a high proliferation index (Ki-67 of 80%). Additionally, decreased positivity for (F) GFAP and (G) increased reticulin deposition are noted in areas with spindle cell morphology. (H, I) Fluorescence in situ hybridization studies confirmed codeletion of chromosomal arms 1p/19q. Scale bar = 20 μm. (J) UMAP demonstrated clustering of the tumor with oligosarcoma reference tumors, closer to mesenchymal glioblastoma, IDH-wild-type, than conventional oligodendroglioma, IDH*-*mutant. Abbreviation: UMAP, uniform manifold approximation and projection.

He underwent repeat temporal craniotomy for resection and diagnostic classification of the extra-axial lesion. Intraoperatively, the tumor was entirely extra-axial and intimately involved with the distal portion of the vein of Labbe. An intraoperative frozen section consultation was consistent with a spindle cell neoplasm, altering the differential to include a meningioma, sarcoma, or a sarcomatous transformation of the patient’s known oligodendroglioma. A gross total resection of this lesion was achieved without complication.

The recurrent lesion underwent histomorphologic and immunohistochemical evaluation, FISH, next-generation sequencing (NGS), and DNA methylation analysis.

The specimen contained a hypercellular, pleomorphic, glial neoplasm with prominent spindle cell morphology and markedly increased mitotic activity (Figure 2B). Neoplastic cells were immunoreactive for IDH1 R132H, increased TP53 staining suggestive of a mutation, and elevated proliferation index with a Ki-67 of 80% (Figure 2C, D, and E, respectively). Areas demonstrating spindled morphology showed decreased GFAP reactivity with increased reticulin deposition (Figure 2F and G, respectively), consistent with a sarcomatous transformation.

1p/19q codeletion in the recurrence was confirmed by FISH (Figure 2H and I). Next-generation sequencing with a 472-gene panel detected mutations in *IDH1* (p.R132H; variant allele frequency (VAF) 31%), *TERT* promoter (C228T, VAF 34%), and *TP53* (p.Y163C; VAF 2% in the setting of loss of captured regions of chromosome 17), with no evidence of *CDKN2A/B* deletion. DNA methylation analysis revealed that the tumor was classified as an oligosarcoma, IDH-mutant, with a high confidence score of 0.9986 (Heidelberg CNS Tumor Methylation Classifier version 12). Uniform manifold approximation and projection (UMAP) analysis revealed that this patient’s tumor, along with other oligosarcoma reference tumors, clusters in closer proximity to the mesenchymal ­glioblastoma, IDH-wild-type, than to conventional oligodendroglioma, IDH-mutant, reflecting this entity’s mesenchymal transformation (Figure 2J). Oligosarcoma is rare, with a worse prognosis than conventional oligodendroglioma and has no definitive CNS WHO grade under current recommendations.^2^^,^^3^

The diagnosis of oligosarcoma prompted additional therapies. The patient completed proton beam radiation therapy and was started on procarbazine, CCNU (lomustine), and vincristine (PCV) chemotherapy, then switched to CCNU and vincristine due to a hypersensitivity to procarbazine. He remained neurologically intact, and an MRI 6 months after resection showed no evidence of disease progression. One year postoperatively, he developed intermittent diplopia and left-sided headaches. MRI demonstrated a multiloculated cystic lesion with peripheral nodular enhancement and a 5-mm left-to-right midline shift suspicious for radiation necrosis versus disease progression (Figure 3A). He was started on dexamethasone with symptomatic improvement. The imaging, prior pathology of the initial and recurrent tumor, and clinical history were again reviewed at the multidisciplinary neuro-oncology tumor board.

**Figure 3.**
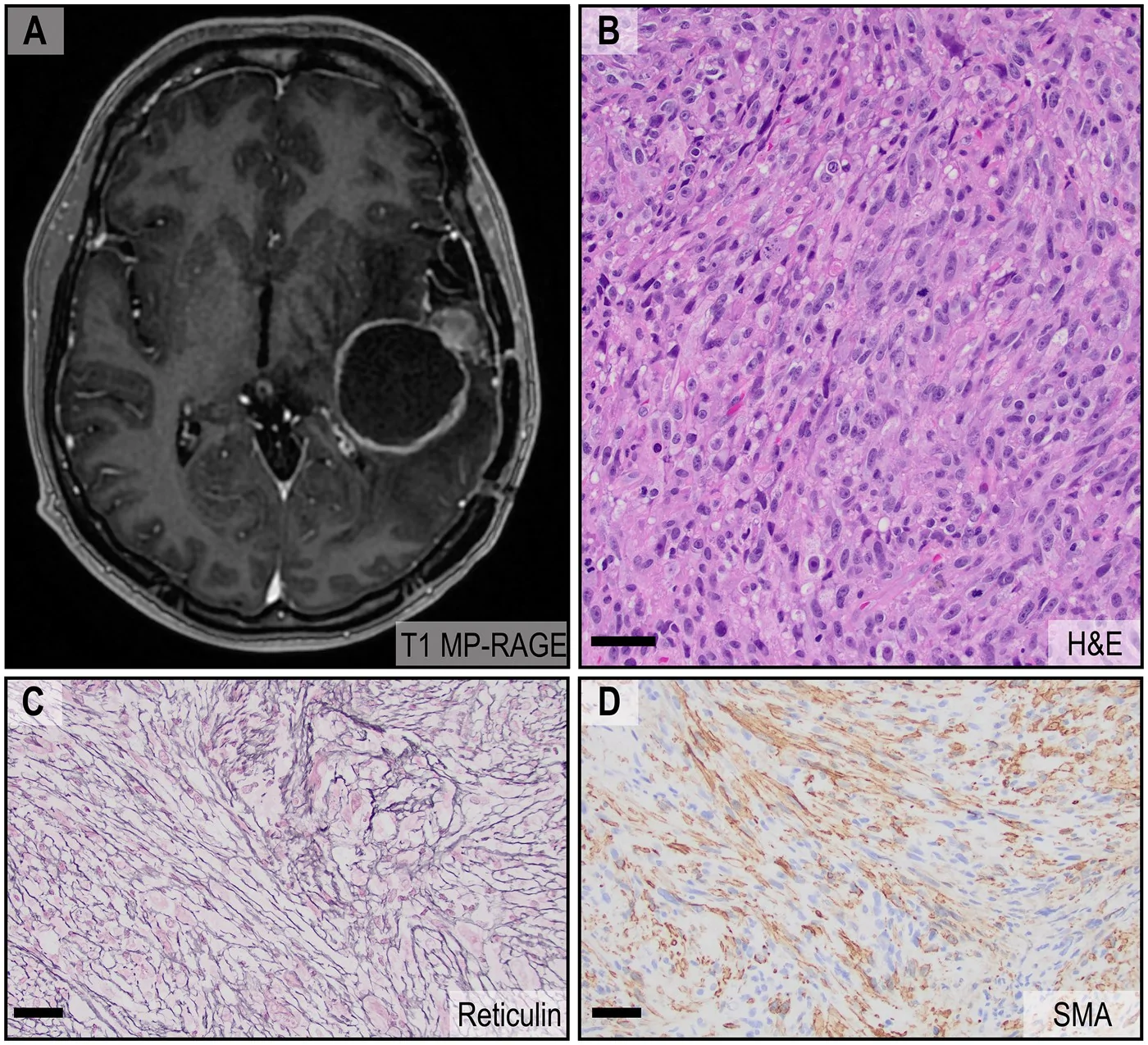
Imaging and histology of second recurrence of an oligodendroglioma with sarcomatous transformation. (A) Axial T1 MP-RAGE MRI of a cystic lesion with nodular area of enhancement and midline shift. (B) Hematoxylin and eosin (H&E) staining of the second recurrence again demonstrates a highly cellular, mitotically active, spindle cell neoplasm with marked nuclear pleomorphism. The neoplastic cells demonstrate (C) increased reticulin deposition and (D) immunopositivity for smooth muscle actin (SMA). Scale bar = 20 μm.

He underwent repeat left temporal craniotomy for resection. Intraoperative frozen section consultation revealed recurrent/residual oligosarcoma. Permanent histology similarly revealed a hypercellular, pleomorphic, glial neoplasm with prominent spindle cell morphology and markedly increased mitotic activity (Figure 3B), consistent with the prior resection specimen. Additionally, immunohistochemical stains were consistent with the prior: immunoreactive for IDH1 R132H, increased TP53 staining suggestive of a mutation, and elevated proliferation index with a Ki-67 of 70%-80%. Areas with spindled morphology showed decreased GFAP reactivity with increased reticulin deposition (Figure 3C) and immunoreactivity to the mesenchymal marker smooth muscle actin (Figure 3D), consistent with residual/recurrent oligosarcoma. Next-generation sequencing was performed on the second recurrence with detected mutations in *IDH1* (p.R132H, VAF 25%), *TERT* promoter (C228T, VAF 44%), and biallelic inactivation of *TP53* (p.F113V, VAF 66% with loss of heterozygosity), similar to the prior recurrence. However, the lesion now demonstrated a subclonal biallelic inactivation of *CDKN2A/B*, representative of clonal evolution.

While there are no standard criteria for the designation of high tumor burden for oligosarcoma due to its rarity, this patient’s second recurrence demonstrated elevated tumor mutational burden (TMB) estimated at 9 mutations per megabase with no evidence of microsatellite instability or mutations in mismatch repair genes by NGS or IHC. IDH-mutant diffuse gliomas typically have a low TMB at diagnosis, with most harboring fewer than 5 mutations per megabase.^4^ Hypermutation is generally restricted to a subset of recurrent tumors following temozolomide, radiation therapy, or a combination of the 2.^5^ Additionally, chemotherapy can increase TMB.^5^^,^^6^ In this case, the patient had not received prior temozolomide but had undergone treatment with chemotherapy and radiation, raising the possibility that these treatments contributed to the observed increase in TMB.

Postoperatively, temozolomide and bevacizumab therapy were initiated, and the patient demonstrated stable imaging findings 4 months later.

## Discussion

Sarcomatous transformation of oligodendroglioma is exceedingly rare, with few cases described in the literature. Oligosarcoma is a histologically biphasic neoplasm with a sarcomatous component arising within or replacing an oligodendroglioma.^2^^,^^3^ Although oligodendrogliomas are defined by IDH-mutation and 1p/19q codeletion and generally demonstrate relatively indolent behavior with favorable responses to chemotherapy and radiation, malignant progression and/or dedifferentiation can occur.^2^^,^^7^

Sarcomatous transformation has been reported most frequently in astrocytomas and glioblastomas, often in the setting of prior radiation or alkylating chemotherapy, and is associated with increased proliferative activity, mesenchymal differentiation, and poor clinical outcome.^8-10^ However, transformation of oligodendroglioma to oligosarcoma remains poorly understood, and its relationship to therapeutic interventions has yet to be established.

Vorasidenib, an oral, brain-penetrant dual inhibitor of mutant *IDH1* and *IDH2*, represents a major advance in the treatment of IDH-mutant diffuse gliomas. By suppressing production of the oncometabolite 2-hydroxyglutarate (2-HG), IDH-inhibition promotes restoration of normal epigenetic and metabolic regulation, delaying tumor progression and reducing the need for subsequent cytotoxic therapy in patients with residual or recurrent grade 2 gliomas.^1^^,^^11^^,^^12^ As clinical use expands, mechanisms of disease progression and therapeutic resistance during or after IDH-inhibition remain incompletely characterized.

In this case, documented radiographic progression occurred while the patient was receiving placebo in the INDIGO trial. Following trial unblinding, he crossed over to vorasidenib and remained on therapy for approximately 1.5 years before developing an enlarging extra-axial lesion diagnosed as oligosarcoma. Although the precise timing of transformation cannot be determined, this chronology suggests that initiation of vorasidenib after established disease progression does not necessarily prevent ongoing aggressive clonal evolution. This observation is particularly relevant as vorasidenib is increasingly incorporated into the management of patients with progressive IDH-mutant gliomas outside the original INDIGO trial population.

Histologically, the recurrent tumors demonstrated high mitotic activity, pleomorphic spindle cell morphology, loss of conventional oligodendroglial architecture, diminished GFAP expression, increased reticulin deposition, and expression of smooth muscle actin, consistent with mesenchymal dedifferentiation. Molecular studies demonstrated persistent *IDH1* mutation and 1p/19q codeletion with *TP53* mutation and subsequent acquisition of subclonal biallelic *CDKN2A/B* inactivation, supporting progressive clonal evolution rather than emergence of an unrelated neoplasm.

The mechanisms underlying this transformation remain speculative. Potential explanations include selection of a resistant subclone with mesenchymal potential, acquisition of additional driver alterations, or therapy-related evolutionary pressures. Experimental studies have demonstrated that IDH-inhibition alters tumor metabolism and epigenetic regulation,^11-13^ while accumulating evidence suggests that these changes may influence tumor evolution under therapeutic selective pressure.^5^ While this single case cannot establish a causal relationship between IDH-inhibition and sarcomatous transformation, it illustrates that malignant evolution may continue despite targeted therapy once progression has occurred.

This case provides a rare longitudinal view of the molecular evolution of an IDH*-*mutant oligodendroglioma into an oligosarcoma. The primary tumor demonstrated the canonical molecular profile of oligodendroglioma, with an *IDH1* mutation and 1p/19q codeletion. At recurrence, the tumor retained these defining alterations while acquiring features of aggressive transformation, including biallelic inactivation of *TP53,* marked mesenchymal differentiation, and, at the second recurrence, subclonal biallelic *CDKN2A/B* inactivation. Rather than representing a biologically distinct neoplasm, these findings support stepwise clonal evolution of the original oligodendroglioma toward an increasingly aggressive sarcomatous phenotype.

Clinically, this case underscores the importance of maintaining a broad differential diagnosis when new or atypical enhancing lesions develop during IDH-inhibitor therapy. Such lesions should not automatically be attributed to expected disease progression or treatment-related change. In this patient, repeat surgical sampling demonstrated a fundamentally different tumor biology that directly altered diagnosis, prognosis, and management. Given the rarity of oligosarcoma and its morphologic overlap with other spindle cell neoplasms, integrated histopathologic, molecular, and DNA methylation profiling remains essential for accurate classification.

## Conclusion

We report a rare case of oligosarcoma arising from an IDH-mutant, 1p/19q-codeleted oligodendroglioma following documented disease progression and subsequent treatment with vorasidenib. The longitudinal clinicopathologic and molecular findings illustrate progressive clonal evolution underlying sarcomatous transformation while emphasizing that atypical radiographic progression during IDH-inhibitor therapy warrants careful evaluation and, when appropriate, repeat tissue sampling. As IDH-targeted therapies become increasingly integrated into clinical practice, continued reporting and comprehensive molecular characterization of similar cases will be critical for defining mechanisms of resistance to IDH-targeted therapies and understanding the natural history of malignant transformation of IDH-mutant gliomas.

## Data Availability

Not applicable.
